# Applicability of the Filter Paper Technique for Detection of Antifilarial IgG_4_ Antibodies Using the Bm14 Filariasis CELISA

**DOI:** 10.1155/2010/594687

**Published:** 2010-02-10

**Authors:** Hayley M. Joseph, Wayne Melrose

**Affiliations:** Lymphatic Filariasis Support Centre, School of Public Health, Tropical Medicine and Rehabilitation Sciences, James Cook University, Townsville, QLD 4811, Australia

## Abstract

Demonstration of successful elimination of lymphatic filariasis (LF) in endemic countries requires sensitive diagnostics for accurate definitions of endpoints and future surveillance. There has been interest in complementing available diagnostics with antibody serology testing in children, since negative serology would correspond with cessation of LF transmission. The Filariasis CELISA detects antifilarial IgG_4_ and has favourable results with serum samples but field application requires an easier sampling method. Ninety-four paired plasma and filter paper samples were assayed with promising results. The filter paper method resulted in a sensitivity of 92% and a specificity of 77% when compared to the paired plasma. One hundred and one filter paper samples were assessed for storage effects. Following 10-month storage at −20^*°*^C there was a significant reduction in reactivity (*P* < .001). Overall the results indicated that filter paper sampling would be a favourable sensitive and specific alternative for blood collection in surveys.

## 1. Introduction

The global program to eliminate lymphatic filariasis (GPELF) currently implements two diagnostic tools: detection of microfilariae (mf) and the detection of circulating filarial antigen (CFA). CFA can be detected by the ICT rapid test or the Og4C3 ELISA. Both tools have proven to be extremely useful during the elimination program stages, where parasite prevalence is relatively high, but there is concern that they might not be sensitive enough to detect residual endemnicity or resurgence in the postprogram phase [1, 2]. Of particular concern is the slow-evolving nature of the disease since individuals may not become CFA or mf positive until up to 12 months post infection [3]. Filarial antibodies develop in response to exposure to the parasite and in endemic countries occur during the first few years of life [4]. In the past, antibody assays in endemic areas were not useful, since the community was constantly exposed and thus antibody positive. With the approach of the end of the mass drug administrations (MDAs), the age prevalence should have shifted, with younger individuals born after the MDA theoretically antibody negative if there has been a cessation of LF transmission. Therefore, it has been suggested that antibody serology be included in the repertoire of LF diagnostics [1]. 

 The LF research community has witnessed the move from earlier antibody assays, reliant on crude parasite lysates, to the recent more specific and sensitive recombinant antigen-based antibody assays (for review see [5]). The earlier assays based on crude parasite lysate were limited in terms of specificity [6, 7], making their use in LF programs inadequate. The advent of recombinant antigen detection systems has increased the specificity of the antibody assays by reducing cross-reaction with other parasitic diseases [8]. The recombinant antigens commercially available are the Bm14, WbSXP, and the BmR1 [8]. 

 The applicability of the recombinant antigens differs depending on the endemic country. BmR1 is a *Brugia malayi *recombinant antigen, which has been shown to react specifically with sera from *B. malayi*-infected individuals [9, 10]; however its specificity for *W. bancrofti* has been reported as quite low [8]. The use of the recombinant antigen Bm14 in the commercially available Filariasis CELISA (Cellabs Pty Ltd., Manly, Australia) combats this problem. 

 The Bm14 assay is an IgG_4_-specific ELISA whereby the plates are coated with the recombinant Bm14 antigen (Cellabs Pty Ltd, Australia). The Bm14 gene belongs to a family of genes encoding proteins that are strong immunogens [11] and was originally isolated from a cDNA library in 1992 for its potential application in LF diagnostics [12]. These initial studies demonstrated the affinity of antibodies isolated from microfilaraemic individuals for the expressed recombinant antigen [12, 13]. The recombinant antigen has been demonstrated to react with sera from patients with brugian or bancroftian filariasis with reported sensitivities of 96% and 91%, respectively, and no cross reaction was reported with 19 serum samples from *Strongyloides* patients [8]. Unfortunately the assay reacted with 72% of the *Loa loa* and *Onchocerca volvulus* positive sera limiting its usefulness in African regions [8]. 

 Although the Bm14 antibody assay has been commercially available from Cellabs, Pty Ltd Australia as a diagnostic tool since 2006, application to large population sizes in field studies has not been thoroughly assessed. Concerns about cross-reactivity in population's endemic for other Helminth parasites, similar to earlier antibody assays, as well as interlaboratory variation have been raised. However, there have been many studies utilising the Bm14, in a research laboratory-based ELISA format, with favourable results [14–17]. The commercially available Filariasis CELISA anti-Bm14 IgG_4_ assay differs slightly from the original prototype research-based ELISA. However, a recent multicentre evaluation has shown promising results with serum (Weil et al., in press) but for any long-term survey work an easier sampling method would be required since venous collection, transportation, and storage of serum can be difficult in endemic areas. Filter paper sampling is more cost-effective, easier, and there has been reports of limited sample variation due to fluctuations in temperature since specimens thoroughly dried can be stable at room temperature for up to a week [18]. Samples could be collected by filter paper method, which has been shown to be a suitable alternative for antifilarial IgG_4_ antibody assays based on crude protein lysate [19]. Filter paper studies for the recombinant antigen Bm14 in the Filariasis CELISA are yet to be ascertained, and The aim of this study was to assess the efficacy of filter paper sampling in a *W. bancrofti* endemic country.

## 2. Materials and Methods

The research was carried out in three different geographical areas. Areas of low LF prevalence were chosen for comparison of sampling techniques and for the effect of storage temperature on filter paper samples. The research was conducted under the human ethics approval numbers H1423 and H2816, as approved by the James Cook University Research Human Ethics Committee.

### 2.1. Study Population

#### 2.1.1. Negative Controls for Filter Paper Sampling

Forty-five nonendemic volunteers, irrespective of age and gender, from Townsville, Australia were selected as negative controls. These individuals had no history of LF exposure or travel to endemic countries. Blood was donated following informed consent.

#### 2.1.2. Antifilarial IgG_4_ in Plasma and Eluates from Filter Paper

Ninety four individuals in the South Pacific country Tuvalu were randomly selected irrespective of age, gender, or previous LF test results. Prior to blood collection verbal consent was given for participation. The study was conducted with the assistance of the Ministry of Health, Tuvalu.

#### 2.1.3. Effect of Storage Temperature on Reactivity of Filter Paper Samples

Following verbal consent, 495 participants in the village of Siufaga in Samoa were screened for antifilarial IgG_4_ antibodies using the Filariasis CELISA (Filariasis CELISA, Cellabs Pty Ltd., Manly, Australia). Screening was performed using the filter paper technique. Based on these preliminary findings, 200 samples were chosen for storage at −20°C for 10 months and retested. The 200 samples chosen were based on the initial optical density (OD) value, whereby a sample was considered reactive if the OD reading was ≥0.400, as per manufacturer's instructions. One hundred and one of the chosen samples were reactive, where 50 samples were high reactors with an OD reading > 1.1. The remaining 51 of the reactive samples had lower OD readings ranging from 0.400 to 0.611. The 99 nonreactive samples chosen ranged from OD readings of 0.0125 to 0.3535. Fifty one of the nonreactive samples had initial OD readings close to the positive cut-off value of ≥0.400 in order to determine if after storage these nonreactive samples became false positives. Results, including OD absorbance values, were compared between the two time periods. The study protocol was reviewed by the Samoan Ministry of Health for approval prior to commencing the research and carried out with the assistance of World Health Organisation, Samoa.

### 2.2. Blood Collection

#### 2.2.1. Negative Controls for Filter Paper Sampling

Filter paper used for collection was the Tropbio filter paper disc (Tropbio Pty Ltd, QLD, Australia), with six protrusions that specifically soak 10 *μ*l of blood each. Blood was collected by fingerprick method directly onto the six protrusions of the filter paper. Filter paper was stored at −20°C until tested.

#### 2.2.2. Antifilarial IgG_4_ in Plasma and Eluates from Filter Paper

Blood was collected from the same individual for a paired plasma and filter paper sample. Approximately 200 *μ*l of blood was collected using the fingerprick method by capillary action into EDTA vacutainers (BD biosciences, Becton, Dickinson and Company, North Ryde, NSW, Australia). Following collection, six x 10 *μ*l blood was blotted onto filter paper, using a micropipette, and left to dry overnight. The remaining blood in the vacutainer was left overnight at 4°C to allow red cell sedimentation. The following morning, the plasma was aliquoted into a fresh sterile tube. Both filter papers and plasma samples were stored at 4°C and transported back to Australia at 4°C at the conclusion of the study. Upon arrival to the Australian laboratory, both filter papers and plasma were stored at −20°C until tested.

#### 2.2.3. Effect of Storage Temperature on Reactivity of Filter Paper Samples

Blood was collected by fingerprick and soaked directly onto each of the 6 protrusions of the filter paper. Following collection, filter papers were left to thoroughly dry, then placed in ziplock bags, and transported back to Australia. Filter papers were stored at −20°C until tested.

### 2.3. Elution of Dried Filter Blood Spots

Sample diluent was prepared according to manufacturer's instructions and 500 *μ*l was transferred into separate serum tubes using a micropipette. A single blood-soaked protrusion was excised into the serum tube, which was then vortexed to ensure complete saturation of the disc. As each protrusion soaks exactly 10 *μ*l, it was assumed that half of this volume constituted serum. Diluting 5 *μ*l of serum in 500 *μ*l of sample diluent resulted in a 1 : 100 dilution. The sample was left to elute overnight at 4°C. The following day the eluates were warmed to room temperature (RT) before commencing the assay. RT was defined as 20°C to 25°C. Samples were thoroughly vortexed prior to testing.

### 2.4. Filariasis C-Enzyme Linked Immunosorbent Assay

Antifilarial IgG_4_ antibodies were detected using the commercially available Filariasis CELISA kit (Cellabs Pty Ltd, Manly, Australia) and samples were tested in duplicate. Initial sample incubation was for 2 hours at 37°C, incubation with secondary IgG_4_ conjugate was for 45 minutes at 37°C, and the final incubation with tetramethylbenzidine (TMB) substrate was in the dark at RT for 15 minutes. The washing steps between incubations were performed with an automated plate washer (MultiDrop Combi nL, Pathtec, VIC, Australia) using 200 *μ*l of washing buffer per well. 

 On completion the OD of the samples was measured at a dual wavelength of 450 nm/650 nm with a Multiskan EX Type 355 Primary V.2.1-0 (Pathtec, VIC, Australia) using the software Labsystems Genesis Version 3.00 (Pathtec, VIC, Australia). Background absorbance of sample diluent was subtracted. Cut-off values differed depending on the sample type. Filter paper samples were considered positive if the OD value is ≥0.400. Serum samples have been shown to have a lower cut-off value for positivity of OD >0.250 [20].

### 2.5. Statistical Analysis

All analyses were performed using the statistical software SPSS Version 17.0. The chi-squared test, with the Kappa agreement statistic, was used to analyse the sensitivity, specificity, positive predictive value (PPV), and the negative predictive value (NPV) of the filter paper sample compared to the gold standard. Ninety-five percent confidence intervals (CI) were reported. Differences in OD readings, between sampling techniques and following storage, were analysed using the Mann-Whitney *U* test.

## 3. Results

### 3.1. Negative Controls for Filter Paper Sampling

**Figure 1:**
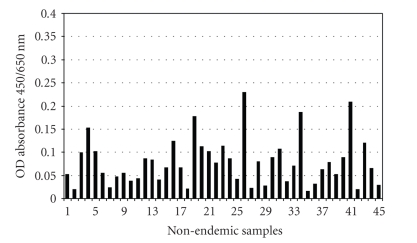
Reactivity of eluates from 45 individuals living in a nonendemic LF area. All of the samples had OD absorbance values lower than the positive cut-off  value of  0.400 and were all considered nonreactive. The average OD absorbance value was 0.08.

The average OD absorbance value obtained for the 45 nonendemic samples was 0.08, and thus well below the positive cut-off value for reactivity of ≥0.400 (Figure 1). Thus there were no reactive samples eluted from filter paper.

### 3.2. Antifilarial IgG_4_ in Plasma and Eluates from Filter Paper

The gold standard was considered to be plasma. There was a significant correlation between the two methods of sampling using the Kappa agreement statistic (*r* = 0.90; *P* < .01), although there was a significantly higher average OD absorbance value for filter paper samples (0.550) than serum (0.401) (*Z* = 6.273; *P* < .001).

In comparison to the gold standard, the sensitivity, specificity, PPV, and NPV for filter paper sampling are summarised in Table 1. The filter paper technique reported a sensitivity of  92% (95%-CI 75–99), specificity of  77% (95%-CI 65–86), PPV of  60% (95%-CI 43–75), and an NPV of  96% (95%-CI 87–100).

### 3.3. Effect of Storage Temperature on Reactivity of Filter Paper Samples

The initial maximum and minimum OD values of the filter paper samples, prior to storage, were 3.9 and 0.013 respectively, with a median of 0.415. Following 10 months of storage at −20°C, the OD maximum and minimum values decreased to 3.5 and 0, respectively, with a median drop to 0.08. This loss of reactivity was significant by Mann-Whitney *U* test (*Z* = 10.9; *P* < .001).

Following 10-month storage, 67 of the original 101 reactive samples dropped below the reactivity cut-off value of ≥0.400 and were deemed nonreactive (Table 2). The remaining 34 reactive samples stayed reactive following storage. All of the 99 nonreactive samples remained nonreactive.

## 4. Discussion

To preserve integrity of samples for sero-epidemiological studies it is important to implement not only the correct collection method but also the correct storage prior to testing. Thus serum collection in endemic countries is often infeasible, not only for difficulties concerning sample preservation but also for cumbersome large-scale sampling that is required. There are many advantages for collecting blood by fingerprick including: less invasive, less patient side effects such as reduced risk of haematoma, and reduced risk of needlestick injury to collector. The reduced risk of needlestick injury is particularly important in areas where night bleeding is still required, or areas where there is potential transmission of blood-borne infectious diseases such as human immunodeficiency virus (HIV) [21]. Collecting the blood onto filter paper would provide a favourable alternative. It is an easy sample to collect and also can be easily obtained from individuals where venous collection is difficult such as children [19]. Another favourable aspect of filter paper sampling is that it has been found in other neglected tropical disease (NTD) programmes, such as the onchocerciasis programme, that introduction of filter paper techniques has increased the number of volunteers willing to participate in blood collection, again contributing to economical feasibility and ease of surveying [21]. 

 The present study clearly demonstrates the applicability of filter paper sampling for detection of antifilarial IgG_4_ antibodies using the Filariasis CELISA. Although filter paper sampling requires overnight elution, the sample preparation is quicker and easier. Filter paper sampling requires submerging the protrusion in sample diluent prior to elution, whereas serum samples are diluted by pipetting. This can be cumbersome when large sampling is involved. Compared to the gold standard, filter paper sampling had excellent sensitivity of 92%, only missing 2 positive serum samples. The specificity was lower (77%) as 16 samples tested positive by filter paper but negative by plasma. This dropped the PPV to be 60%; so when testing a sample, it could possibly be a false positive 40% of the time. The NPV was also excellent (96%) making filter paper sampling quite robust for accurately testing for nonreactive status. 

 The lower specificity, coupled with a low PPV, means that filter paper sampling may result in false positives at an approximate rate of 40% (95%-CI 25% to 57% of the time). Controlled laboratory experiments, utilising blood spiked with a known amount of antibody onto filter paper, showed no significant differences between serum samples and filter paper samples [20]. There could be a number of reasons for the higher OD readings for filter paper samples in the field setting. The sample eluates could contain interfering proteins from the blood. However, addition of a blocking step for nonspecific proteins did not alter the results (data not shown). Another explanation could be the dilution factor. It was assumed that 5 *μ*l of serum was eluted from 10 *μ*l of blood. If the individual was anaemic with a low haematocrit, which can be common in LF endemic countries, the serum to whole blood ratio would increase and potentially more than 5 *μ*l could be eluted. Eluting a higher volume of serum in an antifilarial IgG_4_ positive individual would alter the OD readings of the assay. Further studies would be required to ascertain the cause of the higher reactivity observed in the field setting. 

 Forty percent of samples potentially being false positive (a low PPV) could be disadvantageous for diagnostic testing. However, from a programmatic perspective, the low PPV should not impact greatly on survey work since antifilarial IgG_4_ prevalence rates would be compared annually. Any increase in antifilarial IgG_4_ prevalence rates would flag a problem, rather than the individual's results. If follow-up studies in problem areas required individual diagnostic results, serum samples could be used for confirmation. The high sensitivity and NPV are the crucial aspect for sero-epidemiological studies since a high percentage of false negatives would be more detrimental to the LF program. Therefore, the high NPV and sensitivity observed in this study when using filter paper sampling are advantageous for the LF program. However, until the question of alleged false positivity has been resolved, filter paper test results should be regarded with caution. 

 The decrease in sensitivity observed following 10 months of storage was in agreement with previous studies, which detected antibodies against *Onchocerca volvulus* [21]. In the previous study a significant decrease in antibody detection was observed following 7 months of filter paper storage at −70°C, −20°C, 4°C, and RT. Therefore, the results from the current study suggest that in order to detect individuals with low antifilarial IgG_4_ titres, filter paper testing should occur within the first 10 months of storage. The filter papers utilised in the previous study were the Whatman No.2 papers, which may be less robust than the Tropbio filter paper discs. To ascertain the effect of storage of blood-soaked Tropbio filter paper discs, further storage studies need to be conducted looking at several time points and storage conditions, including −70°C, −20°C, 4°C, and RT. 

 In conclusion, the filter paper collection technique for the detection of antifilarial IgG_4_ antibodies by the commercial kit Filariasis CELISA is a feasible option for future sero-epidemiological surveys. If serology is pursued as part of the LF diagnostic repertoire, filter paper sampling in endemic countries would be more cost-effective and less laborious than venepuncture techniques. For filter paper sampling, the high NPV, coupled with high assay sensitivity, would be advantageous as LF prevalence drops in endemic countries, adding to the usefulness of the assay in post-MDA surveys or future surveillance work. Future work to ascertain the robustness of the assay in large-scale filter paper sampling in an endemic country would be required, including further evaluating the efficacy of storage of filter papers. Overall, filter paper sampling would be more cost-effective and easier than venepuncture and is a favourable alternative method for detection of antifilarial IgG_4_ during survey work in LF endemic areas.

## Figures and Tables

**Table 1:** Cross-tabulation results for the Filariasis CELISA comparing paired plasma and filter paper samples. The gold standard was considered to be the result derived from the plasma sample (columns). ELISA specifications for a filter paper eluate was found to be a sensitivity of 92% (95%-CI = 75–99), specificity of 77% (95%-CI = 65–86), positive predictive value (PPV) of 60% (95%-CI = 43–75), and negative predictive value (NPV) of 96% (95%-CI = 87–100).

		Plasma		
		Negative	Positive	Total
Filter paper	Negative	52	2	54
	*NPV*	96% (52/54)		
	*Specificity*	77% (52/68)		
	Positive	16	24	40
	*PPV*		60% (24/40)	
	*Sensitivity*		92% (24/26)	
	Total	68	26	94

**Table 2:** Cross-tabulation results for the Filariasis CELISA comparing reactivity from paired filter paper samples following 10-month storage at −20°C. Following storage of filter paper samples for 10 months, the reactivity of the samples reduced (*P* < .001). Sixty-seven samples became nonreactive following storage dropping the sensitivity to 34% (95%-CI 25–44) and the negative predictive value (NPV) to 60% (95%-CI 52–67). Specificity (100%) and the positive predictive value (PPV) (100%) remained unchanged following storage (95%-CI 96–100 and 95%-CI 90–100 resp.).

		Initial Result		
		Negative	Positive	Total
Following storage	Negative	99	67	166
	*NPV*	60% (99/166)		
	*Specificity*	100% (99/99)		
	Positive	0	34	34
	*PPV*		100% (34/34)	
	*Sensitivity*		34% (34/101)	
	Total	99	101	200

## References

[1] Weil GJ, Ramzy RMR (2007). Diagnostic tools for filariasis elimination programs. *Trends in Parasitology*.

[2] Huppatz C, Durrheim D, Lammie P, Kelly P, Melrose W (2008). Eliminating lymphatic filariasis—the surveillance challenge. *Tropical Medicine and International Health*.

[3] Sasa M (1976). *Human Filariasis: A Global Survey of Epidemiology and Control*.

[4] Gao CL, Cao WC, Chen XX (1994). Changes in anti-filarial antibody after control of filariasis in Shandong Province. *Chinese Medical Journal*.

[5] Melrose WD, Durrheim DD, Burgess GW (2004). Update on immunological tests for lymphatic filariasis. *Trends in Parasitology*.

[6] Maizels RM, Sutanto I, Gomez-Priego A, Lillywhite J, Denham DA (1985). Specificity of surface molecules of adult Brugia parasites: cross-reactivity with antibody from Wuchereria, Onchocerca and other human filarial infections. *Tropical Medicine and Parasitology*.

[7] Muck AE, Pires ML, Lammie PJ (2003). Influence of infection with non-filarial helminths on the specificity of serological assays for antifilarial immunoglobulin G4. *Transactions of the Royal Society of Tropical Medicine & Hygiene*.

[8] Lammie PJ, Weil G, Noordin R (2004). Recombinant antigen-based antibody assays for the diagnosis and surveillance of lymphatic filariasis—a multicenter trial. *Filaria Journal*.

[9] Rahmah N, Lim BH, Khairul Anuar A (2001). A recombinant antigen-based IgG4 ELISA for the specific and sensitive detection of *Brugia malayi* infection. *Transactions of the Royal Society of Tropical Medicine & Hygiene*.

[10] Rahmah N, Taniawati S, Shenoy RK (2001). Specificity and sensitivity of a rapid dipstick test (Brugia Rapid) in the detection of *Brugia malayi* infection. *Transactions of the Royal Society of Tropical Medicine & Hygiene*.

[11] Rao KVN, Eswaran M, Ravi V (2000). The Wuchereria bancrofti orthologue of *Brugia malayi* SXP1 and the diagnosis of bancroftian filariasis. *Molecular and Biochemical Parasitology*.

[12] Dissanayake S, Xu M, Piessens WF (1992). A cloned antigen for serological diagnosis of Wuchereria bancrofti microfilaremia with daytime blood samples. *Molecular and Biochemical Parasitology*.

[13] Chandrashekar R, Curtis KC, Ramzy RM, Liftis F, Li B-W, Weil GJ (1994). Molecular cloning of *Brugia malayi* antigens for diagnosis of lymphatic filariasis. *Molecular and Biochemical Parasitology*.

[14] Weil GJ, Kastens W, Susapu M (2008). The impact of repeated rounds of mass drug administration with diethylcarbamazine plus albendazole on bancroftian filariasis in papua new Guinea. *PLoS Neglected Tropical Diseases*.

[15] Weil GJ, Ramzy RM, El Setouhy M, Kandil AM, Ahmed ES, Faris R (1999). A longitudinal study of Bancroftian filariasis in the Nile Delta of Egypt: baseline data and one-year follow-up. *American Journal of Tropical Medicine and Hygiene*.

[16] Tisch DJ, Bockarie MJ, Dimber Z (2008). Mass drug administration trial to eliminate lymphatic filariasis in Papua New Guinea: changes in microfilaremia, filarial antigen, and Bm14 antibody after cessation. *American Journal of Tropical Medicine and Hygiene*.

[17] Mladonicky JM, King JD, Liang JL (2009). Assessing transmission of lymphatic filariasis using parasitologic, serologic, and entomologic tools after mass drug administration in American Samoa. *American Journal of Tropical Medicine and Hygiene*.

[18] Coltorti E, Guarnera E, Larrieu E, Santillan G, Aquino A (1988). Seroepidemiology of human hydatidosis: use of dried blood samples on filter paper. *Transactions of the Royal Society of Tropical Medicine & Hygiene*.

[19] Terhell AJ, Haarbrink M, Abadi K (1996). A filter paper technique for the detection of anti-filarial IgG_4_ in lymphatic filariasis. *Transactions of the Royal Society of Tropical Medicine & Hygiene*.

[20] Curtis KC, Fischer PU, Won KY (2009). A multi-centre trial of a new antibody test kit for lymphatic filariasis employing recombinant *Brugia malayi* antigen BM-14. *American Journal of Tropical Medicine and Hygiene*.

[21] Rodriguez-Perez  MA, Danis-Lozano R, Rodriguez MH, Bradley JE (1999). Application of an enzyme-linked immunosorbent assay to detect antibodies to *Onchocerca volvulus* on filter-paper blood spots: effect of storage and temperature on antibody decay. *Transactions of the Royal Society of Tropical Medicine & Hygiene*.

